# Chinese Herbs Medicine Huatan Huoxue Prescription for obstructive sleep apnea hypopnea syndrome as complementary therapy: A protocol for a systematic review and meta-analysis

**DOI:** 10.1097/MD.0000000000021070

**Published:** 2020-07-24

**Authors:** Min Zhou, Qijun Liang, QiuLan Pei, Fan Xu, Hang Wen

**Affiliations:** a College of Clinical Medical, Jiangxi University of Traditional Chinese Medicine; b Affiliated Hospital of Jiangxi University of Traditional Chinese Medicine, Nanchang; c College of Clinical Medical, Shanghai University of Traditional Chinese Medicine, Shanghai, China.

**Keywords:** Chinese medicine, Huatan Huoxue prescription, meta-analysis, protocol, systematic review

## Abstract

**Objective:**

The aim of this systematic review and meta-analysis is to assess effectiveness and safety of Chinese Herbs Medicine Huatan Huoxue Prescription (HTHXP) as complementary therapy in treating bronchiectasis.

**Methods:**

The following databases will be searched: Embase, Cochrane, PubMed, China National Knowledge Infrastructure, Wan Fang, and VIP database from their inception to April 1, 2020. We performed and completed meta-analysis and methodologic evaluation by Review Manager 5.3.3 and statas 12.0 software. Study selection, data extraction, quality assessment, and assessment of risk bias will be performed by 2 reviewers independently. Odds ratios and correlative 95% confidence intervals will be calculated to present the association between the HTHXP and western medicine treatment using Review Manager version 5.3 when there is sufficient available data.

**Results:**

The results will be disseminated through a peer-reviewed journal publication.

**Conclusion:**

These systematic review findings will summarize up-to-date evidence for that HTHXP is more effective and safe as adjunctive treatment for patients with bronchiectasis.

**Ethics and dissemination:**

Ethics approval and patient consent are not required as this study is a systematic review based on published articles.

**PROSPERO registration number:**

INPLASY202050079.

## 1 Introduction

Obstructive sleep apnea hypopnea syndrome (OSAHS) is a common and potentially dangerous sleep disorder accompanied by cardio-cerebrovascular events and multiple organ injury, which is caused by airway occlusion during sleep secondary to pharyngeal collapse and is characterized by repetitive breathing interruptions when sleeping.^[1,2]^ The incidence of OSAHS is ∼2% to 4% in adults worldwide; nevertheless, relevant epidemiologic studies have revealed that the incidence of OSAHS is increasing especially in younger, which is more common in men than in women.^[3–5]^ In recent years, considering the high incidence and mortality of OSAHS, the focus in OSAHS has increased in medical institutions and society.^[6,7]^ Recent statistics have demonstrated that the 5-year mortality rate of untreated OSAHS patients is as high as 11% to 13%, and about 3000 mortalities per day related to OSAHS.^[8]^ What is more, excessive daytime sleepiness reduces the quality of life of OSAHS patients. Up to now, advanced scientific treatments on OSAHS have been achieved, including oral drugs and continuous positive airway pressure (CPAP), and surgery.^[9]^ Nevertheless, despite the advanced treatment, OSAHS patients typically show a poor tolerance and compliance.^[10]^ Furthermore, surgery has been associated with potential complications, such as profuse bleeding, cardiopathy, and hypertension, and uncertainty regarding its long-term therapeutic effect.^[11]^ Therefore, traditional Chinese medicine (TCM) is favored by more patients. Additional research is an urgent need for exploring more effective therapeutic strategies for the treatment of OSAHS.

Natural Chinese herbal medicines that exert anti-OSAHS activities have emerged as a potential strategy for the treatment of OSAHS. TCM differs from western medicine in that western medicine adopts strategies that block a single step in a particular process, whereas TCM uses an overall therapeutic approach to treat and prevent inflammatory responses and oxidative stress with the aim of improving the patient's quality of life. Meanwhile, considering the discovery and widespread application of multiple co-activation pathways in the pathogenesis of OSAHS, single targeted therapy seems to be difficult to achieve the desired therapeutic effect in isolation.^[12]^ It is noteworthy to focus on that TCM is a mixture of various herbs, including a variety of active ingredients, which can act on a number of targets simultaneously.

Since the evidence for HTHXP to treat OSAHS is inconclusive, we evaluate the effectiveness of HTHXP on OSAHS through a systematic review and meta-analysis of existing studies for reference and exploration on further clinical treatment.

## 2 Method

The systematic review protocol has been registered on the INPLASY website (https://inplasy.com/inplasy-2020-5-0079/) and INPLASY registration number is INPLASY202050079.

### 2.1 Search strategy

Six databases including Embase, Cochrane, PubMed, China National Knowledge Infrastructure, Wan Fang, and VIP data were searched from their inception to April 1, 2020. If any, we would try to contact the original study authors for the information we need. What is more, we would perform a manual search to track the references of relevant literature. Then we browsed the detail of the abstract and the full text, and selected eligible studies according to the inclusion criteria. The detailed search strategy for PubMed is demonstrated in Table 1. Similar search strategies would be built for other electronic databases.

**Table 1:**
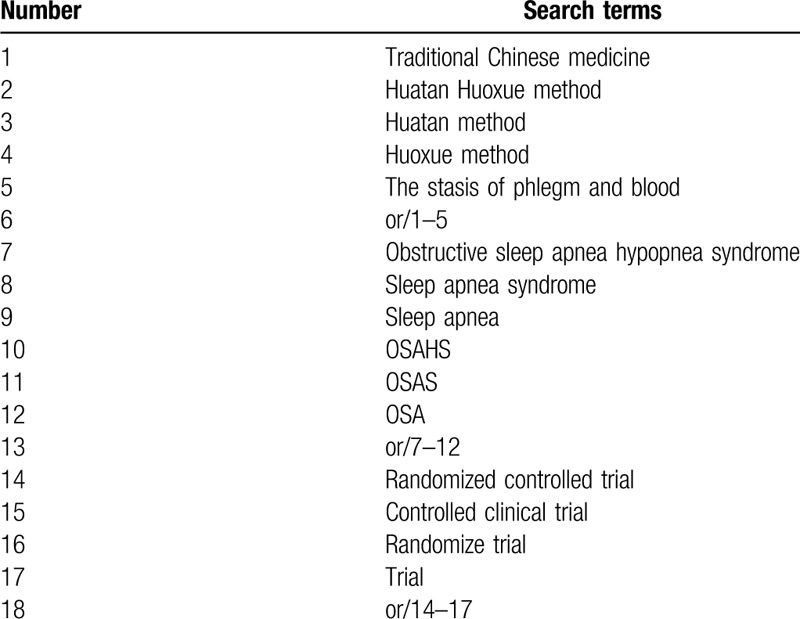
Search strategy for PubMed.

### 2.2 Including and excluding criteria

#### 2.2.1 Including criteria

1. Studies: Clinical randomized controlled trials.
2. Participants: Patients diagnosed with OSAHS according to “2010 Guidelines for the Diagnosis and Treatment of Pulmonary Hypertension in China.”^[14]^
3. Interventions: The treatment group was treated with HTHXM or combined with CWM, and the control group was treated with CWM alone. There was no limitation patient's gender, race, age, and the course and severity of the disease.
4. Outcomes: The effective rate referred to “Guiding principles for clinical research of new Chinese medicine,”^[15]^ AMI, SaO_2_, ESS. Besides, adverse reactions were also took into consideration in this meta-analysis.

#### 2.2.2 Exclusion criteria

1. Studies that were not RCTs
2. Incomplete data literature, and duplicates articles
3. The treatment group was given TCM injections or single Chinese medicine
4. Case reports, reviews, mechanisms, unqualified interventions, and animal model experiments

### 2.3 Data abstraction and quality assessment

**Figure 1:**
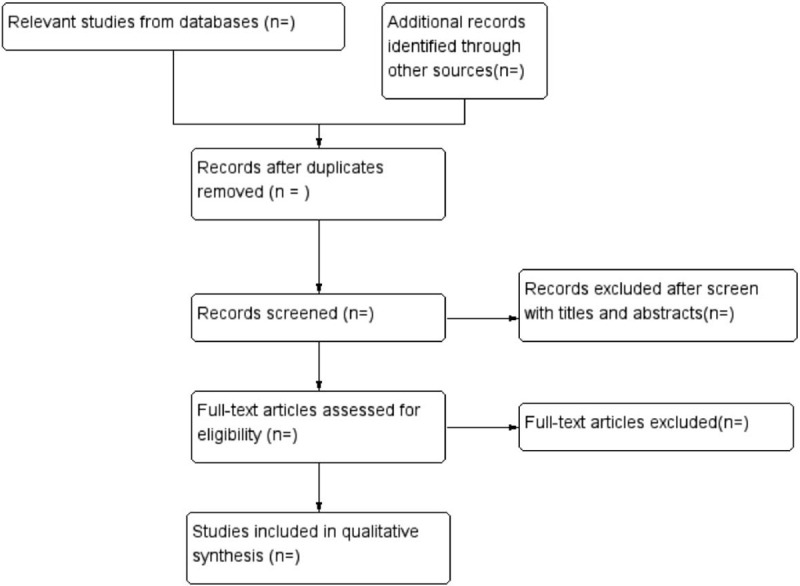
Flowchart of study selection.

Relevant information was extracted and cross checked by 2 independent reviewers (MZ and FX). The extracted data included the first author, sample size, age, interventions details, outcomes, duration, randomization method, blinding of participants and personnel, allocation concealment, incomplete outcome data, follow-up, dropout and withdrawal, and adverse events. If there were disagreements between 2 reviewers, a 3rd reviewer was available to check for accuracy. Details of the selection procedure for studies are shown in a Preferred Reporting Item for Systematic review and Meta-analysis protocol (PRISMA-P) flowchart (Fig. 1).

### 2.4 Quality assessment

The assessment was performed by RevMan 5.3.3 according to the cochrane handbook. The overall assessment were based on details including random sequence generation, blinding of participants and personnel, allocation concealment, incomplete outcome data, selective reporting, and other biases. If we encountered differences, we would discuss with the 3rd reviewer (QL) and finally reach an agreement. The quality assessment was graded as “high” risk, “low” risk, or “unclear” risk depending on the degree of information provided.

### 2.5 Statistical analysis

Statistical analysis was conducted by the Review Manager 5.3 and stata 12.0 software. In this meta-analysis, the mean difference and the odds ratio were adopted to evaluate continuous variable outcomes and dichotomous outcomes with a 95% confidence interval. *P* < .05 was to be of statistical significance. There was no heterogeneity between studies (*I*^2^ < 50%), the fixed effect model was adopted; otherwise the random-effects model was used.

### 2.6 Subgroup analysis

Considering that the treatment effect may be related to the treatment time, the data were analyzed by subgroup according to the treatment time.

### 2.7 Sensitivity analysis

Sensitivity analysis was conducted to evaluate the impact of the included studies on the final outcome.

### 2.8 Publication bias

Forest plots and Egger test was conducted to assess potential publication bias. If *P* < .05, this was considered to be statistically significant. Finally, the Grading of Recommendations Assessment, Development and Evaluation (GRADE) approach was adopted to evaluate the strength of the evidence in order to make our results more convincing.

### 2.9 GRADE quality assessment

The quality of evidence of outcomes will be assessed according to the GRADE system. The GRADE system includes 5 items: the risk of bias, inconsistency, indirectness, imprecision, and publication bias. The quality of evidence will be rated as “high,” “moderate,” “low,” or “very low.”

## 3 Discussion

The OSAHS patients have a special hypoxia pattern at night with high-frequency hypoxic-reoxygenation alternations, which are similar to ischemia-reperfusion. In this process, excessive oxygen-free radicals are generated, which change the balance between oxygenation and antioxidant, leading to the occurrence of oxidative stress. Recent studies have confirmed that oxidative stress markers increase in OSAHS patients, and enhanced oxidative stress is one of the important mechanisms of OSAHS target organ damage. The level of total antioxidant capacity of superoxide dismutase increased, suggesting that TCM can regulate the balance of pro-oxidation and anti-oxidation while improving sleep and respiration.

## Author contributions

**Conceptualization:** Qiulan Pei, Hang Wen.

**Data curation:** Qiulan Pei, Hang Wen.

**Formal analysis:** Min Zhou, Fan Xu.

**Funding acquisition:** Qijun Liang.

**Investigation:** Qiulan Pei.

**Methodology:** Min Zhou, Fan Xu.

**Software:** Qiulan Pei, Min Zhou, Fan Xu.

**Writing – original draft:** Min Zhou, Fan Xu.

**Writing – review & editing:** Min Zhou, Fan Xu, Qijun Liang.
